# Comparison of micro-flow imaging and contrast-enhanced ultrasound in monitoring microwave ablation of papillary thyroid carcinoma: efficacy, safety, and cost-effectiveness

**DOI:** 10.3389/fonc.2025.1545509

**Published:** 2025-08-06

**Authors:** Xiangyu Li, Min Zhuang, Ziyue Hu, Xiaoxiao Xie, Likun Cui, Man Lu

**Affiliations:** Ultrasonography Department, Sichuan Clinical Research Center for Cancer, Sichuan Cancer Hospital & Institute, Sichuan Cancer Center, Affiliated Cancer Hospital of University of Electronic Science and Technology of China, Chengdu, China

**Keywords:** micro-flow imaging (MFI), contrast-enhanced ultrasound (CEUS), microwave ablation (MWA), papillary thyroid carcinoma, monitoring

## Abstract

**Objective:**

To evaluate the value of micro-flow imaging (MFI) in monitoring ultrasound-guided microwave ablation (MWA) of papillary thyroid carcinoma (PTC) by comparing it with contrast-enhanced ultrasound (CEUS).

**Methods:**

This study was conducted on 83 malignant nodules from 79 papillary thyroid carcinoma (PTC) patients treated between June 2022 and September 2023. These cases were divided into a control group (n=37) and an observation group (n=42) based on different guidance modalities during microwave ablation (MWA). The observation group underwent MFI monitoring, while the control group received CEUS monitoring. Clinical parameters were systematically compared between groups, including: 1) baseline clinical characteristics, 2) two-dimensional ultrasound features, 3) vascular patterns (MFI/CEUS), 4) tumor volume (V), 5) volume reduction rate (VRR), 6) postoperative complications, and 7) patient satisfaction outcomes.

**Results:**

All patients successfully underwent ablation. During multiple follow-ups post-MWA, no local recurrence or distant metastasis was observed in either group, and changes in V and VRR were similar (p > 0.05). Consistency was also observed in the number of ablations, ablation time, and postoperative complications between the two groups (p > 0.05). However, the MFI group had lower treatment costs and operation time compared to the CEUS group (p < 0.05), and patients in the MFI group reported higher satisfaction with the procedure (p < 0.05).

**Conclusion:**

The effectiveness and safety of MFI monitoring during PTC ablation are similar to those of CEUS, with the added advantages of lower costs and greater patient satisfaction, making MFI a preferable option for patients.

## Introduction

Thyroid cancer arises from the thyroid follicular epithelium or parafollicular cells and represents the most prevalent malignancy in the head and neck region. Papillary thyroid carcinoma (PTC), constituting approximately 90% of cases, is the predominant histological subtype (1, 2). Conventional management of PTC relies on surgical resection; however, this approach presents clinical challenges, including procedure-related complications, postoperative hypothyroidism necessitating lifelong hormone replacement therapy, and cosmetic concerns associated with scarring (3). In recent years, thermal ablation (TA) has emerged as a minimally invasive alternative for managing benign and malignant thyroid nodules. This technique combines functional preservation, aesthetic advantages, and procedural safety, aligning with patient-centered therapeutic goals. Current evidence supports the clinical efficacy of TA in PTC treatment. The 2021 American Head and Neck Society international consensus guidelines endorse ultrasound-guided TA for select patients with primary papillary thyroid microcarcinoma (PTMC) or recurrent PTC who are ineligible for or decline surgical intervention (4). Precise intraprocedural monitoring is critical to ensuring complete tumor ablation while minimizing complications (5).

The vascular architecture of thyroid malignancies presents unique diagnostic challenges. While thyroid parenchyma is highly vascularized, PTCs typically exhibit avascular characteristics with irregular microvascular patterns (6). Conventional color Doppler ultrasonography, though useful for detecting residual macrovascular flow in ablation zones, demonstrates limited sensitivity for low-velocity blood flow and microvascular structures. Contrast-enhanced ultrasound (CEUS) provides accurate perfusion assessment but carries inherent limitations including invasiveness and elevated costs (7). Consequently, developing non-invasive, cost-effective methods for post-ablation efficacy evaluation could reduce healthcare burdens and expand monitoring options for contrast-contraindicated patients.

Microvascular flow imaging (MFI) employs adaptive motion artifact suppression algorithms to enhance microcirculation visualization, paralleling the capabilities of superb microvascular imaging (SMI) (8, 9). Both modalities have demonstrated clinical utility in microvascular assessment across multiple organ systems. Emerging evidence suggests SMI may effectively detect residual peri-ablation vascularity and quantify ablation volumes, with diagnostic performance comparable to CEUS (10–12). Our prior investigations of MFI in benign thyroid nodule ablation demonstrated equivalent safety and efficacy profiles to CEUS (13). Nevertheless, the clinical validity of MFI/SMI for monitoring TA outcomes in avascular tumors such as PTC remains unestablished. This study aims to evaluate the potential utility of MFI in achieving complete PTC ablation.

## Methods

### Ethical considerations

This study was approved by the Ethics Committee of Sichuan Cancer Hospital (Reference No. SCCHEC-032017-008). All patients participating in the treatment signed informed consent prior to ablation and CEUS.

### Patients

**Figure 1:**
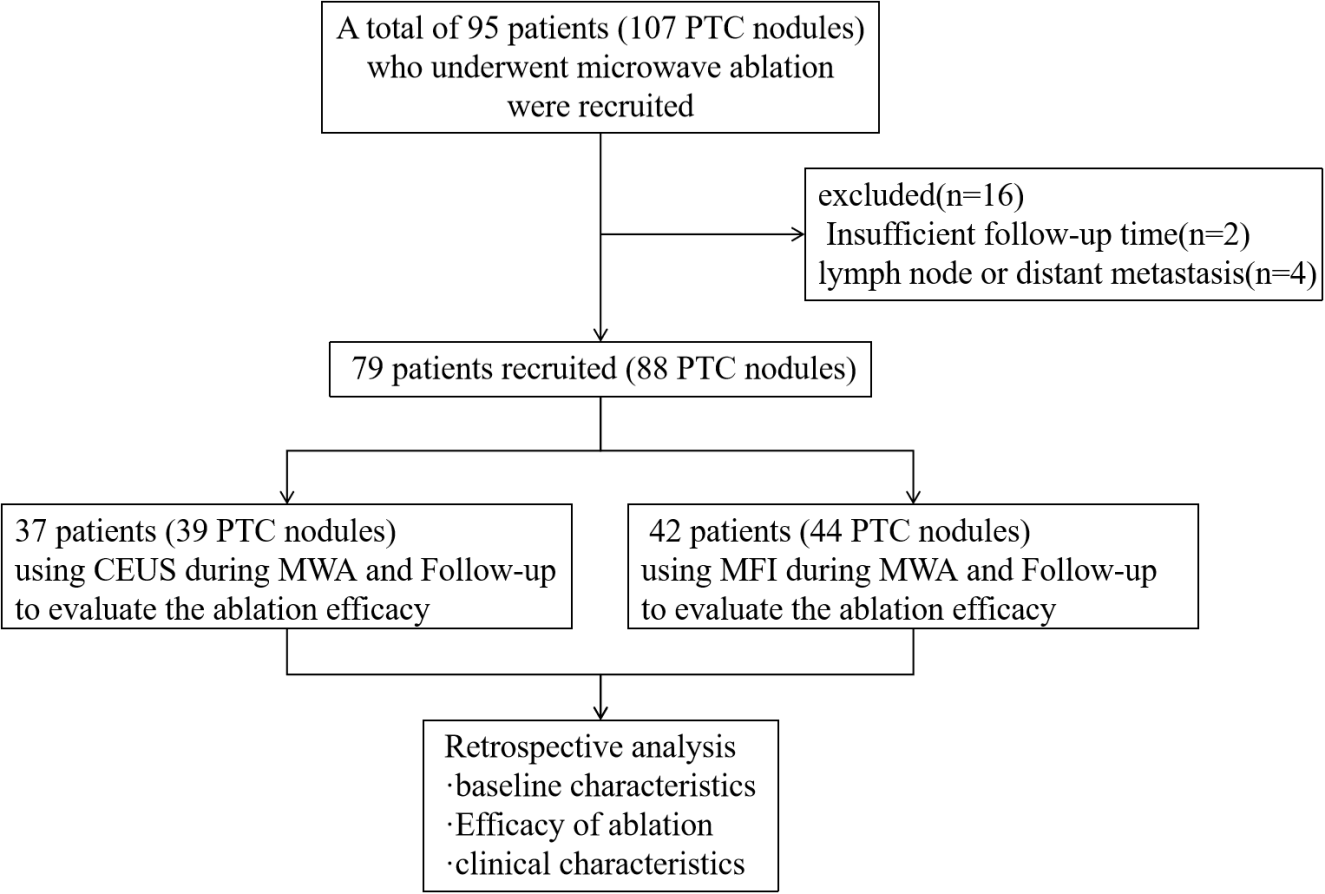
Study flowchart. CEUS, contrast-enhanced ultrasound; MFI, micro-flow imaging; MWA, microwave ablation.

This retrospective cohort study analyzed patients with histologically confirmed PTC who underwent MWA at our institution between January 2022 and September 2024. Prior to intervention, all patients received comprehensive counseling regarding two intraprocedural guidance modalities: MFI and CEUS. Detailed comparisons of technical principles, cost-effectiveness profiles, and clinical applications were provided to facilitate informed decision-making. Based on patient preferences and socioeconomic considerations, participants self-selected their monitoring modality, with no overlap between groups due to cost constraints, two cohorts with different monitoring methods were formed, and we selected samples from these two cohorts according to the principle of randomization. The final cohort comprised 79 patients divided into two arms: a CEUS-guided group (n=37) and an MFI-guided group (n=42). Inclusion criteria required: (i) cytopathological confirmation via fine-needle aspiration (FNA); (ii) documented surgical contraindications or patient refusal of conventional surgery; (iii) no prior thyroid interventions; (iv) a minimum of 9-month follow-up data. Exclusion criteria included: (i) benign histopathology; (ii) incomplete clinical records; (iii) contraindications to ultrasound contrast agents; (iv) confirmed nodal or distant metastases, extrathyroidal extension. The study design is summarized in **Figure 1**.

### Ablation process

Procedures utilized the Philips EPIQ7 ultrasound system (Philips Healthcare, Bothell, WA, USA) equipped with an L12–5 linear array transducer (5–12 MHz) for real-time guidance. Preprocedural evaluations included multimodal imaging (grayscale ultrasound, color Doppler, CEUS, MFI), FNA cytology, and clinical assessments, with documentation of nodule dimensions, anatomical position, and vascular features.

MWA was performed using the KY2000 microwave generator (Kangyou Medical Devices, Nanjing, China) with a 16-gauge internally cooled antenna. Patients were positioned supine with neck hyperextension to optimize thyroid exposure. Following standard antisepsis and local infiltration anesthesia (2% lidocaine), hydrodissection with normal saline established a 5 mm peri-thyroidal protective margin to minimize thermal injury. Under continuous ultrasound guidance, the antenna was percutaneously advanced into the target lesion. Ablation power ranged from 20–60 W, with routine prophylactic ablation of central compartment lymph nodes (level VI), targeting nodal structures that either demonstrated no preoperative ultrasonographic evidence of metastasis or exhibited negative FNA cytology results, but intraoperative imaging guidance revealed discernible lymph node architecture. All procedures were conducted by a board-certified interventional radiologist with >8 years of thyroid ablation experience.

**Figure 2:**
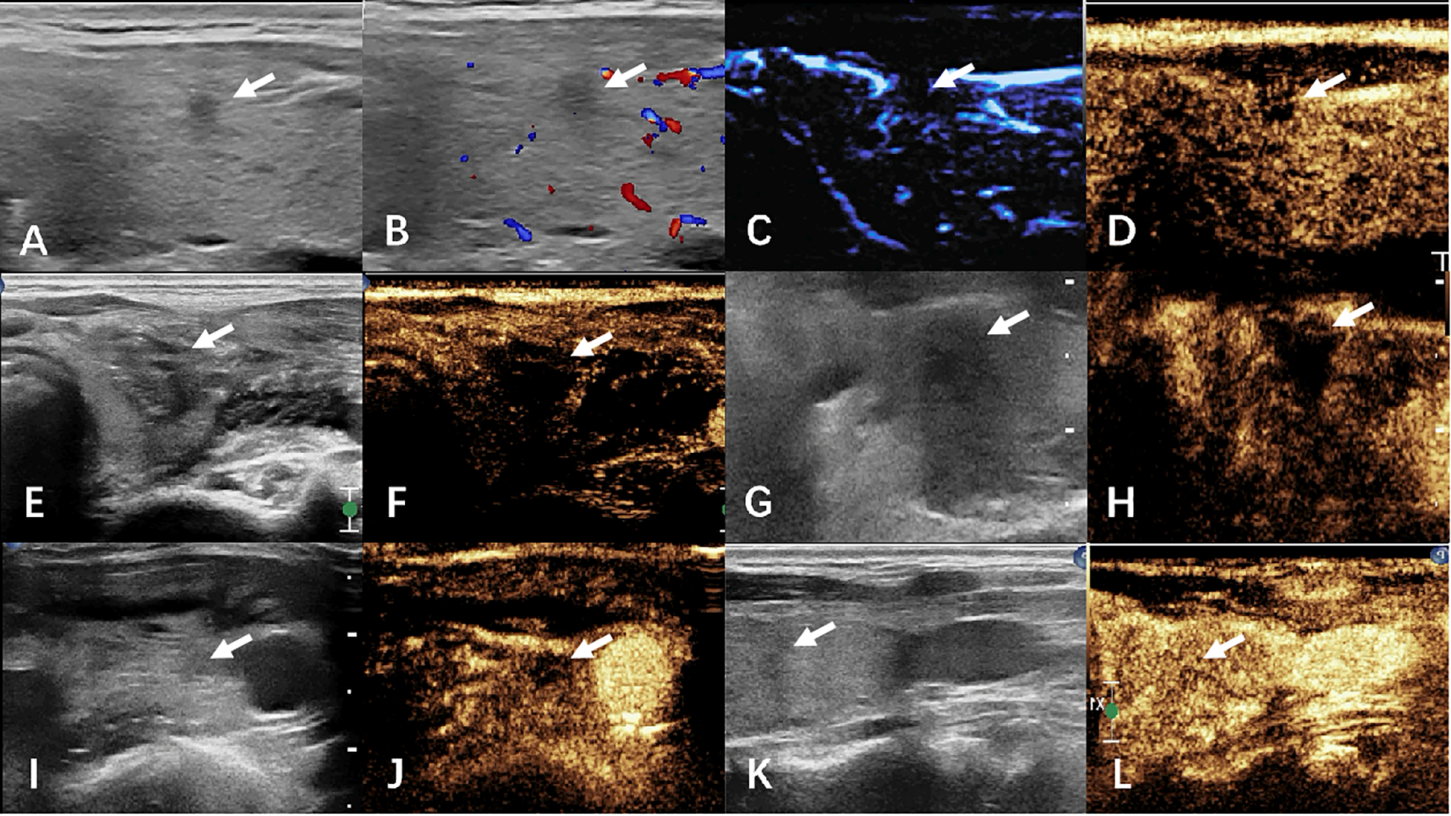
The images of CEUS group before and after ablation were compared. **(A–D)** preoperative ultrasound, CDFI, MFI, and CEUS. **(E, F)** Ultrasound and CEUS immediately after ablation. **(G, H)** ultrasound and CEUS at 1 month after surgery. **(I, J)** Ultrasound and CEUS at 6 months after surgery. **(K, L)** Ultrasound and CEUS at 12 months after surgery. White arrows indicate lesions.

**Figure 3:**
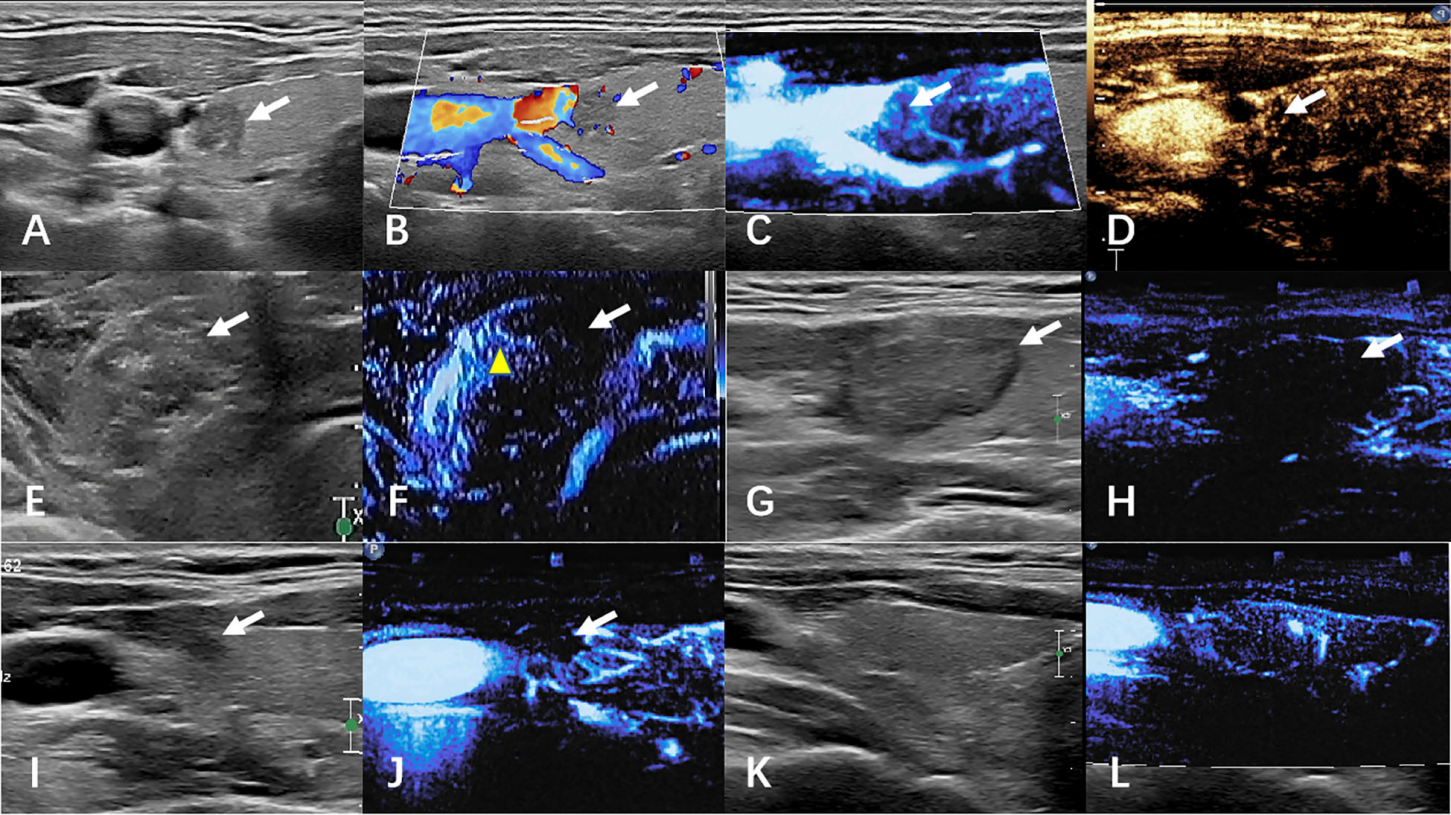
The images of MFI group before and after ablation were compared. **(A–D)** preoperative ultrasound, CDFI, MFI, and CEUS. **(E, F)** Ultrasound and CEUS immediately after ablation. **(G, H)** ultrasound and MFI at 1 month after surgery. **(I, J)** Ultrasound and MFI at 6 months after surgery. **(K, L)** Ultrasound and MFI at 12 months after surgery. White arrows indicate lesions. The yellow arrow shows artifacts caused by gas.

Termination criteria included complete hyperechoic coverage of the target lesion with ≥5 mm ablative margins. Residual tumor detection differed between groups: CEUS identified incomplete ablation via contrast perfusion defects in controls, while MFI detected persistent microvascular flow signals at ablation zone peripheries in the observation cohort. Immediate supplementary ablation was administered for residual lesions, with procedural duration recorded. Technical success was defined as absence of contrast enhancement (CEUS group) or vascular signals (MFI group) post-ablation. **Figures 2**, **3** respectively illustrate the ablation and follow-up processes of the two groups.

### Follow-up examination

Post-ablation surveillance was conducted at scheduled intervals (1, 3, 6, 9, 12, and 18 months) utilizing conventional ultrasound combined with superb microvascular imaging (SMI) and/or contrast-enhanced ultrasound (CEUS) to evaluate thyroid parenchyma and cervical lymph node status. Systematic assessments included: (1) dimensional parameters (maximum diameter and volume) of the ablation zone; (2) sonographic evolution of ablated lesions; (3) clinical symptom progression; and (4) procedure-related complications documented in clinical records. The volume reduction rate (VRR) was calculated as: VRR = (initial volume - final volume)/initial volume × 100%.

### Statistical analysis

Data were analyzed using SPSS 26.0 software (IBM Corp, Armonk, NY, USA). Continuous variables were presented as mean ± standard deviation (mean ± SD) and analyzed using t-tests or one-way ANOVA. Categorical data were expressed as frequencies and analyzed using non-parametric tests. A p-value of < 0.05 was considered statistically significant.

## Results

### Patient baseline characteristics

**Table 1** delineates the baseline parameters of the MFI (n=42) and CEUS (n=37) cohorts. Both groups demonstrated comparable demographic profiles (MFI: 13 males, 29 females; CEUS: 11 males, 26 females) with mean ages of 44 ± 10 vs. 45 ± 10 years (p>0.05). Pretreatment nodule volumes showed no statistical difference (MFI: 281.12 ± 568.52 mm³; CEUS: 279.76 ± 482.98 mm³). TNM staging distribution was comparable between groups (MFI: T1a=31, T1b=9, T2 = 2; CEUS: T1a=24, T1b=10, T2 = 3), with no significant staging disparities (p>0.05). All participants completed ≥12-month follow-up.

**Table 1:** Clinical characteristics of study population.

Characteristic	CEUS group	MFI group	*p*
Sex			*0.91*
Male	11	13	
Female	26	29	
Age (y)	45 ± 10	44 ± 10	*0.70*
Location			*0.77*
Left lobe	17	18	
Right lobe	19	22	
Isthmus	3	4	
Longest diameter (mm)	8.11 ± 3.76	7.81 ± 4.69	*0.53*
Volume (mm^3^)	279.76 ± 482.98	281.12 ± 568.52	*0.89*
T stage			*0.83*
T1a	24	31	
T1b	10	9	
T2	3	2	
Follow-up time (mo)	14.5 ± 3.1	13.8 ± 3.0	*0.23*

CEUS, contrast-enhanced ultrasound; MFI, micro-flow imaging.

### Changes in tumors post-ablation in both groups

Complete technical success was achieved in all index lesions. As detailed in **Table 2**, both modalities demonstrated progressive volume reduction over 18 months. Median pretreatment volumes decreased from 115 mm³ (IQR:10-2932) to 14.66 mm³ (1.57-450.91) in CEUS versus 110 mm³ (13-3267) to 11.38 mm³ (2.17-491.46) in MFI. All nodules achieved >50% VRR, with 68.7% (57/83) demonstrating complete sonographic disappearance (MFI:68.2% [30/44]; CEUS:69.2% [27/39]; p>0.05). Subgroup analysis of T2 lesions (MFI=2, CEUS=3) confirmed equivalent ablation efficacy (**Table 3**).

**Table 2:** Changes of V and VRR between two groups at post-ablation at each follow-up time-point.

Follow-up time (mo)	V (mm^3^)			VRR (%)		
	MFI group	CEUS group	p Value	MFI group	CEUS group	*p*
1	1324.88 (158.22-4486.14)	1250.46 (146.60-4984.63)	0.67	-661.0 ± 583.9	-622.2 ± 412.1	*0.76*
3	467.35 (21.73-1222.53)	398.75 (26.70-1068.10)	0.30	-82.1 ± 71.1	-83.3 ± 88.6	*0.11*
6	93.76 (10.08-958.34)	100.61 (8.90-922.24)	0.13	32.9 ± 14.0	32.5 ± 16.5	*0.62*
9	35.28 (7.06-610.77)	43.98 (7.85-594.22)	0.15	53.0 ± 9.8	52.0 ± 17.1	*0.92*
12	20.33 (4.88-445.92)	26.47 (4.19-414.43)	0.17	74.2 ± 5.4	74.0 ± 10.4	*0.91*
18	11.56 (2.17-249.72)	12.69 (1.57-250.91)	0.51	87.5 ± 3.0	84.7 ± 5.4	*0.29*

CEUS, contrast-enhanced ultrasound; MFI, micro-flow imaging; VRR, volume reduction rate.

**Table 3:** Details of patients with T2 stage.

No. of patients/sex/age (y)	Guiding method	Side	Longest diameter (mm)	Ablation time (sec)	Operative time (min)	Supple-mentary ablation times	VRR at last follow-up(%)	Follow-up period (mo)	Cost (yuan)
1/female/78	MFI	Left	22	915	72.29	1	81.78	18	13195.75
2/female/46	MFI	Left	26	1010	78.42	1	73.14	12	12914.56
3/female/28	CEUS	Right	24	1097	86.94	1	68.29	9	15552.56
4/male/43	CEUS	Right	20	575	71.36	0	89.62	18	14609.26
5/female/30	CEUS	Left	22	789	76.85	1	82.45	12	14966.78

CEUS, contrast-enhanced ultrasound; MFI, micro-flow imaging; VRR, volume reduction rate.

### Comparison of clinical features between groups

As detailed in **Table 4**, the overall complication rate was 11.4% (9/79), with no significant intergroup differences. The CEUS cohort exhibited three complication types: mild pain (n=2), neck swelling (n=4), and transient hoarseness (n=1), while the MFI group demonstrated two categories: pain (n=3) and swelling (n=2). Post-interventional management ensured complete resolution of pain and swelling within 7 days, with hoarseness demonstrating progressive recovery over 3 months. All patients recovered from complications without sequelae. Supplemental ablation was required in 5 cases (CEUS=3, MFI=2), exclusively for lesions exceeding 10 mm (T1b/T2). Procedural efficiency metrics significantly favored MFI, demonstrating reduced total operative duration (49.13 vs. 57.48 minutes; p<0.05) and lower economic burden (¥13,398.33 vs. ¥14,840.46; p<0.05). Patient-reported satisfaction scores were markedly higher in the MFI cohort (p<0.05).

**Table 4:** Comparison of clinical characteristics between two groups.

Characteristic	CEUS group	MFI group	P value
Ablation time (s)	341.76 ± 205.62	332.74 ± 213.87	0.85
Operative time (min)	57.48 ± 10.37	49.13 ± 12.00	<0.05
Supplementary ablation times	3	2	0.27
Complications			0.75
Edema	4	2	
Pain	2	3	
Hoarseness	1	0	
Cost (yuan)	14,840.46	13,398.33	<0.05
Patient satisfaction	7.52	8.34	<0.05

## Discussion

In this study, we compared the monitoring value of Micro-Flow Imaging (MFI) and Contrast-Enhanced Ultrasound (CEUS) during and after the ablation of papillary thyroid carcinoma (PTC) by quantifying volume changes in the ablation zones and analyzing relevant clinical characteristics. The results showed that MFI (n=42) and CEUS (n=37) provided comparable guidance efficacy during PTC ablation. Both methods achieved a 100% technical success rate and equivalent volume reduction. Ultrasonography confirmed the complete disappearance of lesions in 68.7% of cases (p > 0.05), a finding consistent across all TNM subgroups. The incidence and types of complications (11.4%) showed no statistically significant difference between the two groups.

These findings hold clinical significance for selecting monitoring techniques in PTC ablation therapy. PTC, as the most common type of thyroid malignancy (1), is seeing a paradigm shift in its treatment strategy from traditional radical surgery toward minimally invasive ablation techniques. With innovations in image guidance, thermal ablation techniques, represented by microwave ablation (MWA), have become an important alternative therapy for stage T1 PTC due to their precision, repeatability, and low complication rate (3–5). The key to successful PTC ablation lies in achieving complete ablation, which requires reliable monitoring methods to confirm full coverage of the ablation zone (14). However, Color Doppler Ultrasound (CDUS) has limited sensitivity for detecting small vessels and slow blood flow, making it difficult to assess whether the ablated nodule is completely necrotic. Therefore, CEUS is widely used to evaluate ablation efficacy. Its advantage lies in real-time dynamic visualization of tissue perfusion; the absence of contrast agent perfusion within the ablation zone indicates the disappearance of intranodular microcirculation, serving as an indicator of ablation completeness (15, 16).

In this study, all patients in both groups were successfully treated with no recurrence or metastasis observed. During follow-up, the volume change (ΔV) and volume reduction ratio (VRR) of the ablation zones showed high consistency between the groups. At the one-month postoperative assessment, the ablation zone volume was significantly larger than the pre-ablation tumor volume, reflecting adherence to the principle of extensive ablation for malignant tumors. Over time, VRR progressively increased. At the 18-month follow-up, the volume reduction rate was 87.5% ± 3.0% in the MFI group and 84.7% ± 5.4% in the CEUS group, confirming the consistency of both methods in assessing ablation zone volume changes. This complements the prospective study by Lan et al. (12), which demonstrated that different observers using superb microvascular imaging (SMI) to assess ablation completeness showed a linear correlation (r > 0.7) and good consistency (ICC > 0.8) with ablation zone volumes assessed by CEUS. Furthermore, the supplemental ablation rate in this study was 8.1% (3/37) in the CEUS group, slightly higher than the 4.8% (2/42) in the MFI group. This aligns with the findings of Liu et al. (17) regarding the consistency in detection efficacy between SMI and CEUS, where their team reported incomplete ablation detection rates of 14.45% for SMI and 16.02% for CEUS. Notably, cases requiring supplemental ablation in both groups were concentrated in tumors larger than 10 mm in diameter (T1b/T2 stage). This is closely related to the thermal field distribution characteristics of MWA; when the tumor diameter exceeds the effective range of thermal ablation (8–10 mm), “thermal blind zones” are prone to occur at the edges.

While CEUS provides good assessment of tumor ablation efficacy, it has clinical drawbacks such as higher cost and contraindication in patients allergic to contrast agents. MFI technology, utilizing adaptive algorithms to separate motion artifacts from true blood flow signals, demonstrates unique advantages in detecting low-flow microvessels (9, 18, 19). This study showed that the total procedure time in the MFI group was 14.5% shorter than in the CEUS group (p<0.05). This difference is attributed to the additional 5–10 minutes required for CEUS to complete contrast agent injection and monitor the circulation phase. Importantly, MFI demonstrated greater efficiency advantages in patients with multiple nodules (≥3 nodules), saving an average of 18.7 minutes per case. This offers a new strategy for managing complex clinical cases. Regarding economic cost, the per-patient expense in the MFI group was 12.6% lower, primarily due to avoiding contrast agent consumption (each CEUS requires 1–2 vials of SonoVue^®^, costing approximately ¥800 per vial). Costs increase when CEUS assessments are needed more frequently for patients with multiple nodules or requiring immediate supplemental ablation during MWA due to contrast agent usage. The advantages in time cost and economic cost resulted in higher patient satisfaction with ablation therapy in the MFI group compared to the CEUS group (4.7/5 vs. 4.1/5, p=0.03).

Despite MFI’s significant advantages over CEUS in time-cost efficiency, it has inherent limitations in vascular morphological assessment. CEUS can provide more comprehensive hemodynamic information through quantitative analysis of parameters like time to peak (TTP) and peak intensity (PI) (20, 21). In contrast, MFI may still miss microvessels with diameters less than 50 μm. The distinct characteristics of MFI and CEUS lead to differences in clinical selection: CEUS holds advantages in qualitative assessment (e.g., differentiating tumor viability) and quantitative analysis (e.g., hemodynamic parameters), while MFI excels in real-time guidance and cost control (22). Therefore, a combined application of both techniques could be considered in specific clinical scenarios, such as for PTC nodules larger than 1 cm (T1b stage and above) or multiple nodules. A “MFI initial screening + CEUS detailed examination” strategy could be employed, where lesions suspected of residual activity on MFI undergo CEUS confirmation. This multi-angle approach can jointly determine ablation boundaries and zone viability.

This study has certain limitations. First, there are potential confounding factors in the inter-group comparison: although baseline characteristics were balanced (P>0.05), the cohort design implementing MFI and CEUS separately might be influenced by individual microenvironmental differences. For instance, two dorsal thyroid lesions in the CEUS group adjacent to the esophagus showed delayed contrast enhancement, while no similar anatomical interference cases occurred in the MFI group. Future studies could adopt a self-controlled design to reduce heterogeneity between patients. Second, functional assessment was incomplete: this study focused on morphological changes and did not conduct further correlation analysis of hemodynamic parameters (e.g., TTP/PI from CEUS vs. MFI blood flow grading), somewhat limiting in-depth comparison at the functional level. Additionally, while the 18-month follow-up validated short-term efficacy, the small sample size (n=79) limits the power to detect rare complications (e.g., recurrent laryngeal nerve injury), and data on tumor recurrence rates beyond 5 years are lacking. Future large-scale, multicenter prospective studies (n≥500) with long-term follow-up (according to ATA guidelines) are needed to strengthen the evidence.

In conclusion, MFI, as an emerging non-invasive imaging technique, demonstrates monitoring efficacy similar to CEUS in MWA for PTC, while offering lower time costs and better economic efficiency. Furthermore, MFI’s high-frame-rate microvascular imaging complements CEUS’s macro blood flow perfusion assessment. Their combined application could provide multi-dimensional blood flow information support for superficial tumor ablation. Future clinical research will validate its applicability in different types of thyroid lesions and explore its potential value in the ablation of other solid tumors.

## Conclusion

In our study, MFI and CEUS had similar monitoring effects on the process of PTC ablation. While the treatment effect is consistent, the use of MFI not only shortens the operation time, but also reduces the economic cost of the patient.

CEUS, contrast-enhanced ultrasound; MFI, micro-flow imaging.

## Data Availability

The original contributions presented in the study are included in the article/supplementary material. Further inquiries can be directed to the corresponding author.
